# Preclinical pharmacology of AZD9977: A novel mineralocorticoid receptor modulator separating organ protection from effects on electrolyte excretion

**DOI:** 10.1371/journal.pone.0193380

**Published:** 2018-02-23

**Authors:** Krister Bamberg, Ulrika Johansson, Karl Edman, Lena William-Olsson, Susanna Myhre, Anders Gunnarsson, Stefan Geschwindner, Anna Aagaard, Anna Björnson Granqvist, Frédéric Jaisser, Yufeng Huang, Kenneth L. Granberg, Rasmus Jansson-Löfmark, Judith Hartleib-Geschwindner

**Affiliations:** 1 Cardiovascular, Renal and Metabolic Diseases, Innovative Medicines and Early Development Biotech Unit, AstraZeneca, Gothenburg, Sweden; 2 Discovery Sciences, Innovative Medicines and Early Development Biotech Unit, AstraZeneca, Gothenburg, Sweden; 3 Centre de Recherche des Cordeliers, INSERM U1138 Team 1, Paris, France; 4 Division of Nephrology & Hypertension, Department of Internal Medicine, University of Utah School of Medicine, Salt Lake City, Utah, United States of America; University Medical Center Utrecht, NETHERLANDS

## Abstract

Excess mineralocorticoid receptor (MR) activation promotes target organ dysfunction, vascular injury and fibrosis. MR antagonists like eplerenone are used for treating heart failure, but their use is limited due to the compound class-inherent hyperkalemia risk. Here we present evidence that AZD9977, a first-in-class MR modulator shows cardio-renal protection despite a mechanism-based reduced liability to cause hyperkalemia. AZD9977 *in vitro* potency and binding mode to MR were characterized using reporter gene, binding, cofactor recruitment assays and X-ray crystallopgraphy. Organ protection was studied in uni-nephrectomised db/db mice and uni-nephrectomised rats administered aldosterone and high salt. Acute effects of single compound doses on urinary electrolyte excretion were tested in rats on a low salt diet. AZD9977 and eplerenone showed similar human MR *in vitro* potencies. Unlike eplerenone, AZD9977 is a partial MR antagonist due to its unique interaction pattern with MR, which results in a distinct recruitment of co-factor peptides when compared to eplerenone. AZD9977 dose dependently reduced albuminuria and improved kidney histopathology similar to eplerenone in db/db uni-nephrectomised mice and uni-nephrectomised rats. In acute testing, AZD9977 did not affect urinary Na^+^/K^+^ ratio, while eplerenone increased the Na^+^/K^+^ ratio dose dependently. AZD9977 is a selective MR modulator, retaining organ protection without acute effect on urinary electrolyte excretion. This predicts a reduced hyperkalemia risk and AZD9977 therefore has the potential to deliver a safe, efficacious treatment to patients prone to hyperkalemia.

## Introduction

Mineralocorticoid receptor (MR, NR3C2) is a ligand activated transcription factor belonging to the oxysteroid nuclear hormone receptor class. Activation of MR by aldosterone in kidney tubules and collecting duct epithelial cells regulates electrolyte and fluid homeostasis and consequently plays a pivotal role in blood pressure control [1]. MR is also expressed in a wide variety of different non-epithelial cell types and tissues [2–5], where it mediates a large diversity of cell type specific effects. MR activation in non-epithelial tissues promotes target organ dysfunction by stimulating inflammation, oxidative stress and fibrosis [6]. Pathological activation of MR leads to an increased risk for cardiovascular (CV) events [7, 8] and MR blockade using the marketed MR antagonists (MRA) spironolactone and eplerenone has proven to be an effective therapy for treatment of heart failure and hypertension [9–11].

Furthermore, a growing body of pre-clinical and clinical evidence supports a role of MR activation as an independent and powerful mediator of renal vascular disease and chronic kidney disease (CKD) [12]. High levels of circulating aldosterone are associated with endothelial dysfunction [13–16] and can lead to the development of impaired renal function characterized by kidney fibrosis, hyperfiltration and proteinuria [12, 17].

Clinical studies have shown that MRAs on top of angiotensin converting enzyme inhibitors (ACEi) or angiotensin receptor blockers (ARB) further improve proteinuria, have the potential to slow down CKD progression and reduce the number of CV events in CKD patients [12, 18–20]. Several studies suggest that the kidney protective effects of MRAs cannot be explained by blood pressure reduction only. For example, White et al. reduced blood pressure using either eplerenone or the calcium channel blocker amlodipine to the same extent but only eplerenone treatment led to significant reduced urinary albumin/creatinine ratio (UACR) [21].

The use of spironolactone and eplerenone, which antagonize MR similarly in both epithelial and non-epithelial tissues, is limited mainly by the mechanism based risk for hyperkalemia, which is increased 3- to 8-fold in patients with reduced kidney function (CKD stage ≥ 3) [22, 23].

A tissue/cell type specific, selective MR modulator which antagonizes MR in non-epithelial cells but has minimal effect on MR mediated electrolyte handling in epithelial cells has the potential to overcome the limitations of current MRAs and deliver significant therapeutic benefit. Selective nuclear receptor modulators, which act as antagonists for some target gene/tissues and agonists for others, have been developed previously for other nuclear receptors. The partial agonism shared by these compounds allows the receptor to adopt a wider spectrum of structural states, which enables binding of both co-activators and co-repressors depending on the cellular context [24]. For example, selective estrogen receptor (ER) modulators promote ligand specific ER conformations which translate into distinct co-factor recruitment and gene expression patterns [25, 26]. Similar ligand dependent regulation of co-factor recruitment and gene expression has also been demonstrated for the other members of the oxosteroid receptor family to which MR belongs, i.e. the androgen (AR), glucocorticoid (GR) and progesterone (PR) receptors [27–30].

There is limited information available regarding compound selective co-factors for MR and most studies are based upon recruitment of isolated peptide fragments [31, 32]. However, several lines of evidence suggest that development of selective MR modulators may be feasible. For example, MR has two physiological ligands—aldosterone and cortisol. Cortisol has been suggested to be the primary ligand of MR in non-epithelial cells and, dependent on the oxidative state of the cell, may act as MR antagonist or agonist [33]. Cortisol or aldosterone bound MR induces distinct gene expression patterns indicating ligand specific differences [34–36].

The present report describes the pharmacological profile of AZD9977, a novel, non-steroidal selective MR modulator, which separates organ protective effects from acute effects on urinary electrolyte excretion in pre-clinical models. Emerging structural and functional characterization of AZD9977 compared to eplerenone suggests, that AZD9977 has a distinct molecular mode of action compared to classical MRAs like eplerenone.

## Materials and methods

### Protein expression, purification, crystallization and structure determination

The MR ligand binding domain (LBD, amino acids 735–984) with the mutations C808S, C910S to improve expression yields and an N-terminal, TEV cleavable 6-HN tag and a C-terminal thrombin cleavable co-activator peptide PQAQQKSLLQQLLTE was expressed and purified in the presence of ligand as described previously [37]. Crystals were grown using sitting drop vapor diffusion at 293K. The precipitant solutions were 0.9 M (NH_4_)_2_SO_4_, 0.1 M CHES pH 9.5, supplemented with 10% (v/v) of condition E5 from the Morpheus Screen (Molecular Dimensions) for AZD9977 and 10% (v/v) of conditions G5 of the Morpheus Screen (Molecular Dimensions) for eplerenone, respectively. The crystals were dipped into well solution supplemented with 18% ethylene glycol before being flash frozen in liquid nitrogen.

X-ray diffraction data were collected from a single crystal at 100K at ID29 and ID23-1 at the European Synchrotron Radiation Facility (Grenoble, France). Data were integrated and processed using XDS [38] and programs from the CCP4 suite [39]. The MR:eplerenone and MR:AZD9977 crystals belonged to the space groups P212121 (49 78 79 90 90 90) and P21212 (49 78 79 90 90 90), respectively, with one molecule in the asymmetric unit. The structures were solved by molecular replacement using the Protein Data Bank (PDB) entry 4uda [37] as search model. The structure was refined using the BUSTER [40] and manual rebuilding using Coot [41]. The final MR:eplerenone and MR:AZD9977 models had an R_work_/R_free_ of 0.23/0.24 and 0.21/0.24, respectively, with no outliers in the Ramachandran plots. The coordinates and structure factors have been deposited into the PDB with the accession codes 5MWY and 5MWP (entries will be released upon publication). Structural figures were prepared using Pymol [42].

### Ligand binding assays

A scintillation proximity-based radioligand binding assay was used to measure the ligand displacement of aldosterone to human MR-LBD as described previously [37]. Filtration based radioligand binding assays for human GR-LBD (catalogue number 0469), AR-LBD (catalogue number 0933) and PR-LBD (catalogue number 2341) were performed by Eurofins Cerep SA, France.

### Reporter gene assays

To assess ligand activities on full length MR, U2-OS cells (ATCC HTB96^™^) were transiently transfected via electroporation with hMR/pCDNA 3.1 or hMR(M777V)/pCDNA3.1 expression plasmids and a MMTV-luc2P/pGL4.36 reporter plasmid. To determine species specific ligand activities, a reporter plasmid where luciferase is driven by the yeast GAL4 response element was cotransfected with plasmids expressing a fusion protein between the GAL4 DNA binding domain and the human, rat or mouse MR LBD. Transfected cells were seeded in 384 well plates prior to addition of AZD9977 or eplerenone diluted in cell medium (with or without aldosterone) and incubation at 37°C, 5% CO_2_ for 24 hours. Cells were lysed with SteadyLite (Perkin Elmer) according to manufacturers protocol and luminescence was measured using an Envision plate reader. Analysis and curve fitting was performed using GraphPad Prism 6.0 (GraphPad Software, Inc, CA, USA).

### Co-factor recruitment assay using surface plasmon resonance

Co-activator recruitment of MR was determined using surface plasmon resonance (SPR) biosensor (Biacore 3000, GE Healthcare) by probing the level of receptor binding to seven surface-immobilized co-activator peptides. All peptides carried an N-terminal biotin for surface-immobilisation, the following peptides and sequences were used:

CREP binding protein: GSGSGSGNLVPDAASKHKQLSELLRGGSGS; Mediator of RNA polymerase II transcription subunit 1: GSGSGSHGEDFSKVSQNPILTSLLQITGNG; nuclear receptor coactivator (NcoA) 1: GSGSGSSGPQTPQAQQKSLLQQLLTEA; NcoA2: GSGSGSEPVSPKKKENALLRYLLDKDDTK; NcoA3: GSGSGSEQLSPKKKENNALLRYLLDRDDP; peroxisome proliferator-activated receptor gamma coactivator (PRGC) 1: GSGSGSPPQEAEEPSLLKKLLLAPANT; PRGC2: GSGSGSPAPEVDELSLLQKLLLATSYP.

MR-LBD (amino acids 712–984) with the mutations C808S and an N-terminal, thrombin cleavable 6-HN tag were expressed and purified in the presence of 50 μM dexamethasone due to the instability of the apo-receptor, as described previously [37]. Prior to the SPR measurements, excess dexamethasone was removed by gel filtration via a passage through a PD10 column (GE Healthcare) equilibrated in SPR running buffer (10 mM HEPES, 50 mM NaCl, 0,005% P20, pH 7.6). This was followed by an immediate equilibration of the MR-LBD (120 nM) with either aldosterone, AZD9977 or eplerenone (50 μM) prior to the sample injection over a modified biosensor surface. The surface was prepared through immobilization of the biotinylated peptides on a streptavidin chip (GE Healthcare) to levels of 1000±300 RU. The binding level at equilibrium was monitored for all samples including the reference sample that contained remaining traces of dexamethasone. All data was normalized to this reference sample in order to quantify the effects on peptide binding without bias by receptor instability. The surface was regenerated with 0.5% (w/v) SDS after each injection.

### Organ protection in uni-nephrectomised rats on high-salt diet and aldosterone infusion

The study on uni-nephrectomised rats was carried out by PreClinOmics (Indianapolis, Indiana, USA) and the study protocol was approved under the provisions outlined in PreClinOmics IACUC ethical application proposal #2013–184. Male Sprague-Dawley rats (n = 48, 240 to 280 g) underwent surgical uni-nephrectomy (Charles River Laboratories, Portage, MI, USA) prior to arrival. The rats were maintained on Purina 5001 standard rodent chow *ad libitum* during facility acclimation (7 days). Animals were housed 2 rats/cage with a standard 12 hour light cycle and were kept at 22 to 25°C. Immediately after acclimatization, Alzet osmotic pumps (Durect Corp., model 2ML4) were implanted under brief isoflurane anesthesia to deliver aldosterone (Sigma, Cat #A9477, formulated as 3 mg mL^-1^ in 0.15% DMSO/sterile water) subcutaneously at a constant rate of 0.75 μg/hr, throughout the study. Following mini pump implantation, animals were monitored daily for any clinical signs (especially inflammation at the subcutaneous incision site) for 1 week. If any redness was observed at the incision site, the site was cleaned with betadine and a topical antibiotic was applied. Drinking water was supplemented with 0.3% KCl to prevent hypokalemia. Standard diet (5001, Purina Teklad, USA) was admixed with 6% NaCl and including test substances (Teklad #9386, Purina Teklad, USA) for the remainder of the study. Animals were assigned to treatment groups (n = 8) based on body weight before pump implantation. A Mahalanobis Outlier Distance Plot and the T2 statistic was used to identify outlier urinary albumin values within each group after 4 weeks of treatment. One animal each in the vehicle, 100 mg kg^-1^ d^-1^ AZD9977 and 30 mg kg^-1^ d^-1^ eplerenone treated groups were omitted from analysis, final number of animals included in the analyses are indicated in S1 Table.

On the same day as aldosterone/high salt diet began, treatment with either vehicle, AZD9977 or eplerenone (Kemprotech Limited, UK) was started. The test substances were provided in powder form to Purina Teklad for pelleting in Teklad 9386 diet at 0.18, 0.54 or 1.8 g kg^-1^ diet. These concentrations were designed to deliver 10, 30 or 100 mg kg^-1^ d^-1^, respectively. Test diets were fed *ad libitum* for 4 weeks. Animals were placed in standard rodent metabolic cages, on 3 separate occasions, with food and water for collection of 24 hour urine samples at baseline and 2 and 4 weeks after pump implantation. Urine samples were collected at room temperature without additives. Urine volume was measured using a graduated cylinder. Urine creatinine (Beckman, OSR6178) was assayed from fresh sample. Albumin (Alpco rat albumin ELISA kit #41-ALBRT-E01) was assayed from frozen urine. For drug quantification, two blood samples per animal were obtained via tail vein at between 7 am and 7 pm on day 29. For details on time points, see Method section for the pharmacokinetic-pharmacodynamic analysis. Animals were anesthetized on day 30 using CO_2_ inhalation and terminated via induction of pneumothorax. Terminal blood samples were collected by cardiac puncture. Plasma K^+^ levels were determined on a Radiometer ABL 700 blood gas analyser (Radiometer, Copenhagen DK), plasma creatinine was determined by liquid chromatogarphy tandem mass spectrometry (LC-MS/MS). Heart and right kidney were fixed in 10% formalin for histological assessment (Premier Laboratory, Boulder, CO, USA).

Premier provided pathologists scoring of Hematoxylin and Eosin, and Masson’s Trichrome stained slides for renal fibrosis and glomerulonephritis. Each animal had two blocks processed, one for myocardium (not reported here) and one for kidney. All blocks were grossed, processed and had two slides cut from each block: one stained with Hematoxylin and Eosin and one stained with Masson’s Trichrome. For kidney sections, pathology involved glomerular lesions, interstitial inflammation and tubular lesions (collectively, “glomerulonephritis”). Fibrosis, both within glomeruli and within the interstitial space, was scored using the Masson’s Trichrome stain. Average renal pathology scores were calculated as the (renal fibrosis score + renal glomerulonephritis score)/2. All scoring was performed on blinded samples.

### Organ protection in db/db uni-nephrectomised mice

Obese diabetic male db/db mice (BKS.Cg-Dock7^m^ +/+ Lepr^db^/J homozygotes) and their littermate male db/m mice were obtained from the Jackson Laboratory (Bar Harbor, ME, USA). The db/db mice were determined to be diabetic by the vendor on the basis of obesity at approximately 5 weeks of age and were further demonstrated to be hyperglycemic at 7 weeks of age. Mice were subjected to right uninephrectomy at 8 weeks of age to hasten the development of diabetic nephropathy as described previously [43]. Db/m mice that received a uninephrectomy at 8 weeks of age served as the operation control. This study was carried out in strict accordance with the recommendations in the Guide for the Care and Use of Laboratory Animals of the National Institutes of Health. The protocol was approved by the Animal Care Committee at the University of Utah (Permit Number: 13–09001). All surgery was performed under isoflurane anesthesia, and all efforts were made to minimize suffering. Animal health and behaviour was monitored daily by staff with appropriate accreditations and several years of experience handling rodents.

Uni-nephrectomised mice were assigned to treatment groups (n = 10) at 18 weeks of age based on body weights and urine albumine excretion (UAE) as follows: (i) untreated non-diabetic db/m mice as healthy controls; (ii) untreated diabetic db/db mice as a disease control at 18 weeks of age; (iii) untreated diabetic db/db mice as disease control at 22 weeks of age; (iv) diabetic db/db mice treated with AZD9977, 100 mg kg^-1^ d^-1^ mixed with food; (v) diabetic db/db mice treated with eplerenone, 100 mg kg^-1^ d^-1^ mixed with food, (vi) diabetic db/db mice treated with enalapril, 200 mg l^-1^ enalapril in drinking water; (vii) diabetic db/db mice treated with AZD9977 plus enalapril and (viii) diabetic db/db mice treated with eplerenone plus enalapril for 4 weeks from 18 to 22 weeks of age. Water consumption was monitored daily, food consumption was measured weekly to confirm correct dosing of AZD9977 and eplerenone.

Individual animals were lost in all groups during the treatment period, final number of analyzed animals is included in S2 Table. Humane endpoints were used and pre-specified criteria for euthanasia included listlessness, difficulties breathing or moving, eating or drinking cessation, weight loss >20%. Animals were euthanised immediately after humane endpoints were established. Briefly, all non-diabetic db/m mice survived during the experiment period. One to 3 mice in the diabetic groups were lost during the 4 weeks experiment period: 4 animals were euthanised in accordance with humane endpoint pre-specifications, 3 died from respiratory infections and 2 died from bladder stones.

The blood glucose level and glycosylated hemoglobin (Hb_A1C_) levels were monitored in tail blood samples using a blood glucose meter (Glucometer Elite XL, Bayer Healthcare, Elkhart, IN, USA) and the DC 2000+ Hb_A1C_ kit (Bayer Healthcare), respectively, before treatment and at the time of sacrifice. Twenty-four-hour urine was obtained from each mouse after placement in metabolic cages before treatment and at the time of sacrifice. Urine albumin was measured using the DC2000+ microalbumin reagent kit (Bayer Healthcare).

Mice were sacrificed under isoflurane anesthesia. Blood samples were obtained by cardiac puncture for the measurements of plasma compound, K^+^ and creatinine concentrations. Plasma K^+^ levels were determined on a Radiometer ABL 700 blood gas analyser (Radiometer, Copnchagen DK). Plasma creatinine levels were determined by LC-MS/MS. Urine creatinine was measured by the Jaffe method (Creatinine FS, REF117119910021, DiaSys). Kidneys were perfused through the heart with cold PBS and then excised. One piece of renal cortex was fixed in 10% neutralized formalin for histological staining.

Formalin-fixed renal cortex tissues were subsequently embedded in paraffin. Three-micrometer sections were cut from the tissue blocks and stained with periodic acid-Schiff (PAS). The PAS-positive glomerular matrix was quantitated in a blinded fashion by a computer-assisted method as previously described [43]. At least 20 glomeruli from each individual mouse were assessed. The PAS-positive material area in the mesangium was normalized by that of the total glomerular tuft where the percentage of mesangial matrix occupying each glomerulus area was rated. Glomerular sclerosis index was graded from 0 to 4+ as follows: 0 represents no lesion, 1+ represents sclerosis of <25% of the glomerulus, while 2 =, 3+ and 4+ represent sclerosis of 25% to 50%, >50% to 75%, and >75% of the glomerulus, respectively. A whole kidney average sclerosis index was obtained by averaging scores from all glomeruli on one section.

### Electrolyte effects *in vivo*

Experimental procedures to assess electrolytes effects in vivo were approved by the local ethics review committee on animal experiments in the Gothenburg region (EA 141–2010). Male Sprague Dawley rats (Crl:CD(SD), Charles River Laboratories, Germany) were housed together in groups of 5 to 6 rats per cage prior to experiment in rooms with controlled humidity (50% relative humidity) and temperature (21 to 22°C) on a 12 hours light/darkness cycle (lights on at 6 am). Animals had environmental enrichment such as nest pads, nesting box, running ladder and aspen chew sticks.

Rats were fed a low salt diet (R70 with 0.02% NaCl, Lantmännen, Kimstad, Sweden) for 72h prior to dosing to increase endogenous aldosterone levels. Indivuals were randomised on body weight and orally gavaged with vehicle (0.5% HPMC, 0.1% Tween80, 5% Solutol HS15) and/or AZD9977 and/or eplerenone (Molekula Limited, UK) at 7:00 am at the day of the experiment. Immediately following dosing, rats were placed in metabolic cages with free access to drinking water but no food. Urine was collected for 8 hours to determine total electrolyte (Na^+^ and K^+^) content as well as total urinary volume. Urine was collected at room temperature with no additives. Urine was analyzed for Na^+^ and K^+^ content on a Radiometer ABL 700 blood gas analyzer (Radiometer, Copenhagen, Denmark).

### Pharmacokinetic-pharmacodynamic analysis

For the uni-nephrectomised rats on high-salt diet and aldosterone infusion study, plasma was sampled at 7 am, 11 am, 3 pm and 7 pm the day before termination and analysed for drug concentrations by LC-MS/MS. Two plasma samples were obtained per animal at either of the listed time points, yielding in total three or four drug exposure measurements per time point. The observed concentrations were used to estimate average drug exposure (C_*ss*,*average*_) levels over a 24-hour period. To obtain representative drug exposure levels overnight, the 7 am drug samples were back-extrapolated to the 7 pm sample. A non-compartmental analys was applied, using the linear trapezoidal linear interpolation method, on the pooled data to obtain a daily average drug exposure levels (C_*ss*,*average*_) for each dose level. Standard errors of the mean for C_*ss*,*average*_ were calculated for each sampling time point per dose level (four standard standard errors per dose levels) and were afterward averaged and multiplied with the estimated C_*ss*,*average*_ per dose level. The C_*ss*,*average*_ levels were converted to free steady state exposure levels (Cu_*ss*,*average*_ = C_*ss*,*average*_ × unbound fraction) to allow comparison of Cu_*ss*, *average*_ with in vitro potencies (Fold IC_50_ = Cu_*ss*,*average*_ */ In vitro* IC_50_). To assess exposure-response relationships data was visually explored [44].

For the db/db uni-nephrectomised mice study, the terminal samples were used to assess drug levels (C_drug_).. To allow comparison of the exposure levels to *in vitro* mouse MR potencies (Fold IC_50_) the C_drug_ was converted to free drug levels by the unbound fraction in plasma.

In the rat urine electrolyte excretion model, satellite pharmacokinetic animals were used with serial plasma sampling to assess drug exposure levels during the study duration. There were in total 13 and 20 satelite animals for AZD9977 and eplerenone, respectively, with three to four plasma drug samples per animal sampled at 0.16, 1, 3, 8 or 24 hours after dose. Exposure data were analysed using nonlinear mixed effects pharmacokinetic modelling. Based on the final population pharmacokinetic model, the eight hour study window was simulated and mean exposure was retrieved from these simulations. The 90% confidence intervals of the mean exposure levels were obtained by Monte Carlo simulations based on the uncertainty, using the parameter covariance matrix, of pharmacokinetic parameter estimates. 1000 simulations were performed and based on these simulations the 5^th^, and 95^th^ percentiles were retrieved.

To assess if AZD9977 and eplerenone cause pharmacokinetic drug-drug interaction in the rat urine electrolyte excretion model, data were visually explored and a non-compartmental analysis was applied using a linear trapezoidal and linear interpolation. There was no clear trend of pharmacokinetic drug-drug interaction and further analysis was therefore omitted. For all pharmacokinetic-pharmacodynamics analyses Phoenix WinNonlin version 6.4, NMLE version 1.3 (Certara USA, Inc., Princeton, NJ) was used.

### Data and statistical analysis

The data and statistical analysis comply with the recommendations on experimental design and analysis in pharmacology [45]. Data are presented as group average ± SEM except for in vitro studies where SD is indicated. Statistical analysis was by ANOVA testing followed Dunnett’s testing for multiple comparisons using Analyse-it for Microsoft Excel 4.51 (Analyse-it Software, Ltd.). All affinity values were converted to their negative logarithm pActivity (e.g. pIC_50_ or pK_i_) with mol L^-1^ as base unit (e.g. 1 μmol L^-1^ is converted to 6 [log Activity units]).

## Results

### AZD9977 is a partial MR antagonist *in vitro*

**Fig 1:**
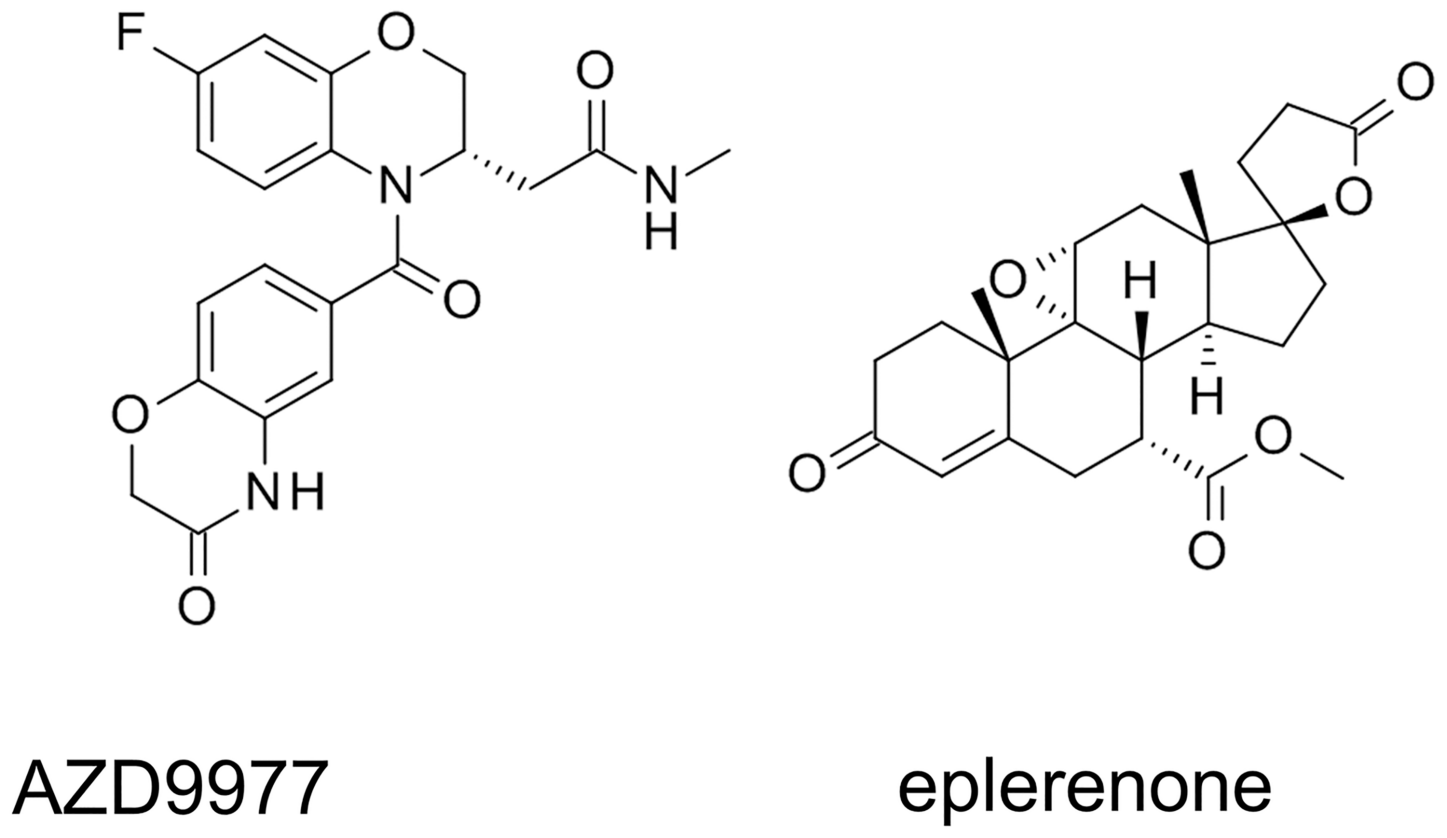
Structures of AZD9977 and eplerenone. Structures of AZD9977 ((S)-2-(7-Fluoro-4-(3-oxo-3,4-dihydro-2H-benzo[b][1,4]oxazine-6-carbonyl)-3,4-dihydro-2H-benzo[b][1,4]oxazin-3-yl)-N-methylacetamide) and eplerenone.

AZD9977 (Fig 1) displaces tritiated aldosterone from recombinantly expressed MR in competition binding experiments. The observed pK_i_ for AZD9977 and the comparator eplerenone was 7.5 and 7.0, respectively, affinities towards GR, PR and AR are listed in Table 1 and reveal similar selectivity profiles of AZD9977 and eplerenone within the NR3C steroid receptor family

**Table 1:** AZD9977 and eplerenone activities on MR, GR, PR and AR in binding assays.

	AZD9977		eplerenone	
	pKi	*n*	pKi	*n*
MR	7.5 ± 0.1	22	7.0 ± 0.1	10
GR	5.4 ± 0.1	3	4.9 ± 0.1	3
PR	4.6 ± 0.4	3	4.3 ± 0.2	3
AR	<4.3; <4.3	2	5.3 ± 0.3	3

Binding constants (pK_i_) ± standard deviations derived from competition binding experiments. n = number of observations

**Fig 2:**
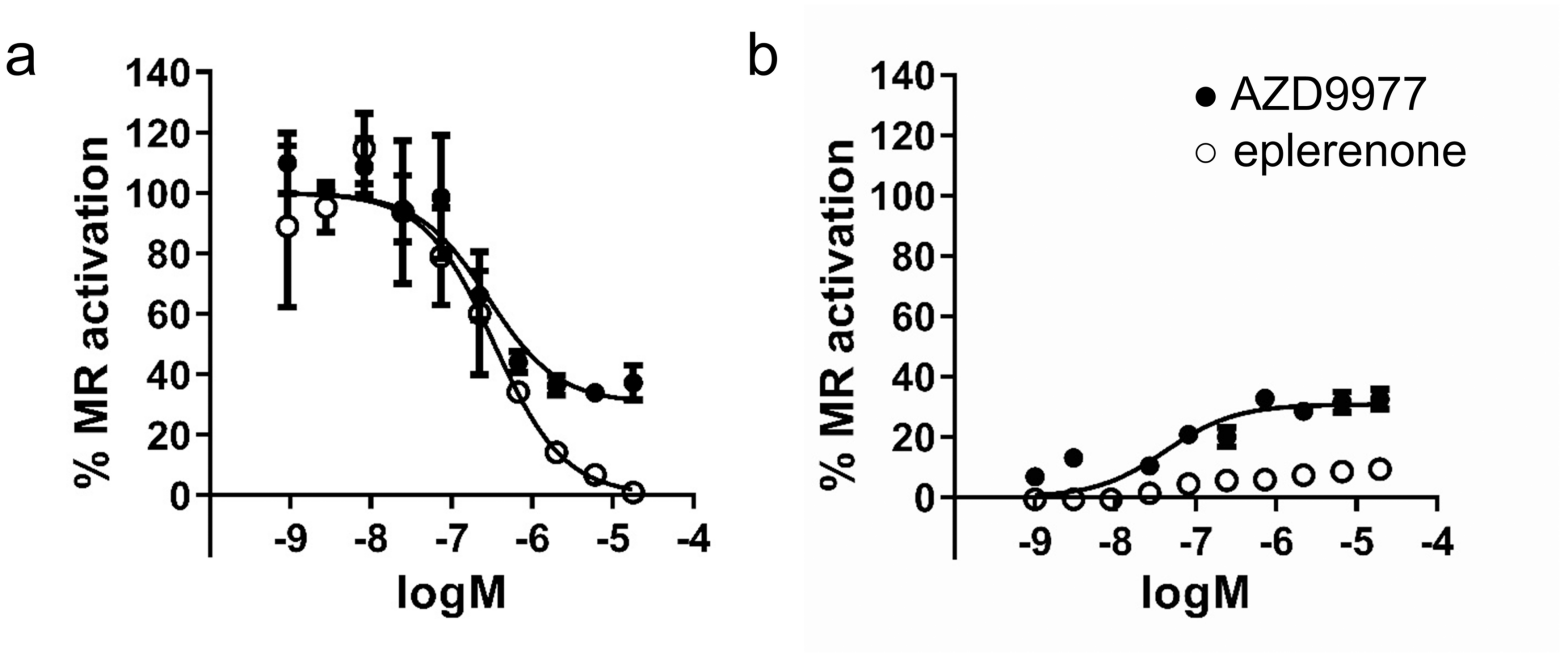
AZD9977 and eplerenone activities in reporter gene assays. Concentration response curves of AZD9977 and eplerenone tested in a reporter gene assay in (a) presence or (b) absence of 0.1 nM aldosterone. *n* = 4, average ± SD.

Functional interaction of AZD9977 with MR was characterized in a reporter gene assay where the full length MR drives a luciferase reporter gene in U2-OS cells. AZD9977 as well as eplerenone antagonise aldosterone activated MR with IC_50_’s of 0.28 and 0.34 μM, respectively. Interestingly, whereas eplerenone is a full antagonist in this assay, AZD9977 suppresses only 69% of the MR activity (Fig 2a). This finding is in agreement with the observation that AZD9977 in absence of aldosterone is a partial agonist with 31% efficacy while eplerenone is inactive (Fig 2b).

Species selective potencies of AZD9977 and eplerenone were established in reporter gene assays using the MR LBDs from human, mouse or rat and corresponding IC_50_ values are listed in Table 2. AZD9977 and eplerenone are equipotent on human and mouse MR, eplerenone is 2-fold more potent on rat MR than AZD9977.

**Table 2:** AZD9977 and eplerenone activities in reporter gene assays for human, mouse and rat MR.

Species	AZD9977			eplerenone		
	pIC_50_	IC_50_ (μM)	N	pIC_50_	IC_50_ (μM)	N
Human	6.4 ± 0.1	0.37	3	6.4 ± 0.2	0.37	5
Mouse	7.1 ± 0.1	0.08	4	7.1 ± 0.1	0.09	4
Rat	7.1 ± 0.1	0.08	8	7.4 ± 0.1	0.04	15

### AZD9977 displays a differentiated binding pattern to MR and cofactor recruitment profile

To develop a molecular understanding of the differential pharmacology observed for AZD9977, the X-ray structures of MR LBD with mutations Cys808Ser and Cys910Ser in complex with AZD9977 and eplerenone were determined. MR exhibits the characteristic three layered alpha helical fold with the ligand binding pocket fully occluded at the center [32, 46, 47].

**Fig 3:**
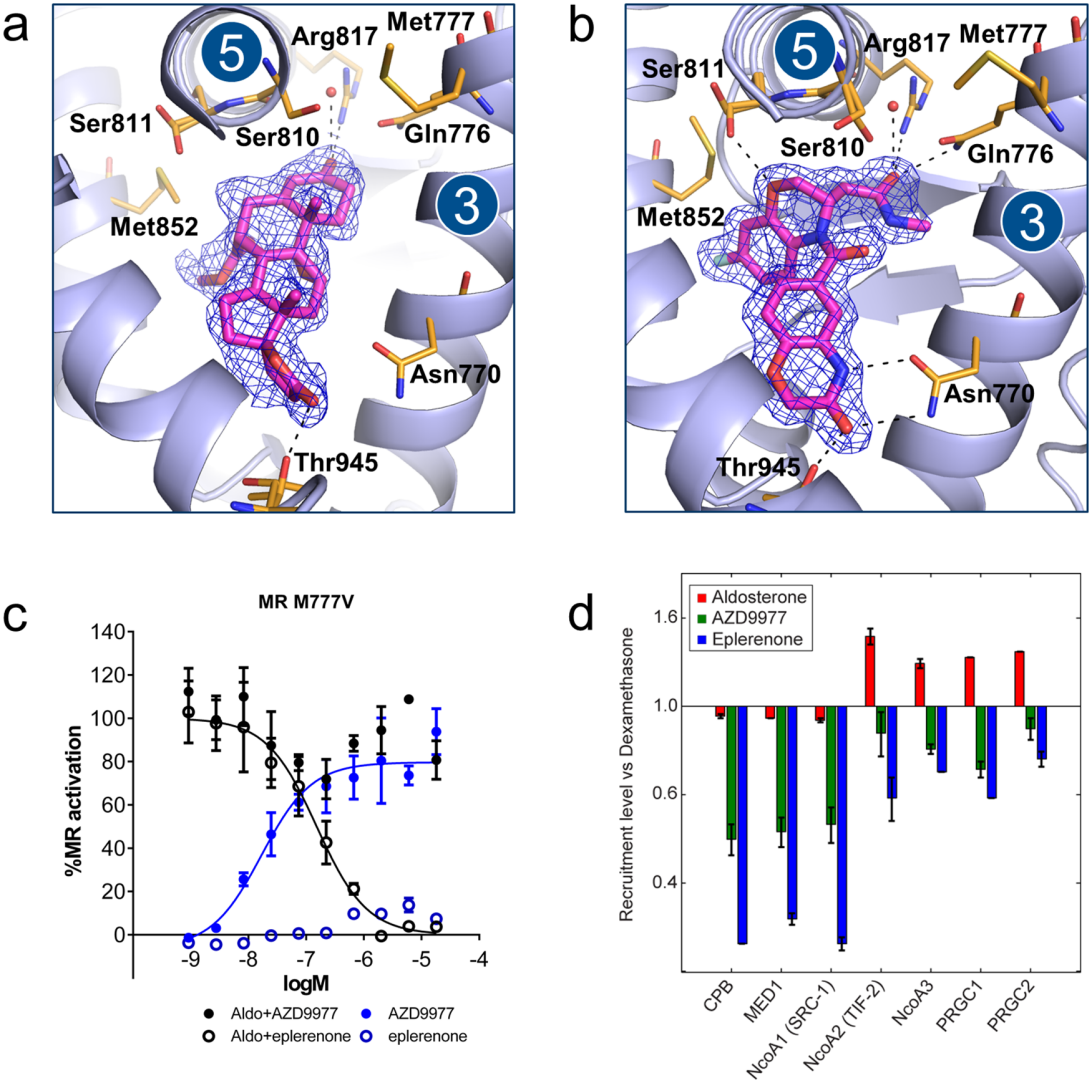
Crystal structure, reporter gene data on M777V mutation and co-peptide recruitment comparing AZD9977 and eplerenone. The structures of the mineralocorticoid receptor (MR) ligand binding domain (LBD) in complex with (a) eplerenone and (b) AZD9977. The ligands are shown in magenta surrounded by refined 2mFo-DFc electron density as indicated by blue mesh. Putative hydrogen bonds are marked as dashed lines. Near the interface in between helices 3 and 5, the AZD9977 amide extension is placed with the oxygen in a position within hydrogen bonding distance of Gln776, Arg817, Ser810 and an ordered water molecule. In contrast, the eplerenone 3-keto group is further removed from the helix 3–5 interface and only appeared to make a direct interaction with Arg817. In addition, the AZD9977 3,4-dihydro-2*H*-1,4-benzoxazine oxygen is positioned to make a direct interaction to one of the observed rotamers of Ser811. As Ser811 is unique to MR within the steroid receptor family, this interaction may contribute to the selectivity profile of AZD9977. At the other end of the ligand binding pocket, the AZD9977 *N*-methyl-acetamide side chain is optimally placed to form a direct interaction to Thr945 and a bidentate interaction with Asn770. While the eplerenone γ-lactone is positioned to interact with Thr945 it is further away from Asn770 and only appears to make a weak interaction with the asparagine Nδ. (c) Reporter gene data for AZD9977 (solid) or eplerenone (open) tested on Met777Val MR mutation in presence (black) or absence (blue) of 0.1 nM aldosterone. *n* = 4, average ± SD. (d) Recruitment, relative to reference compound (dexamethasone), of MR-LBD to seven different co-regulator peptides for aldosterone (green), AZD9977 (red) and eplerenone (blue) at saturated binding conditions (50 μM). *n* = 2, average ± SD.

The binding mode of eplerenone (Fig 3a) was closely related to that of spironolactone in complex with MR (Ser810Leu), crystallized in the absence of coactivator peptide [48]. While AZD9977 exploited the same regions of the ligand binding pocket, it formed a distinct interaction pattern to the receptor (Fig 3b). Notably, AZD9977 had stronger interactions to both the gatekeeper residues (Gln776 and Arg817 at the helix 3—helix 5 interface) and to Asn770. These are both important receptor activation triggers [46] and the observed interaction patterns are in line with the partial antagonist profile of AZD9977.

The distinct interaction pattern between AZD9977 and MR LBD is transmitted to the second shell residues surrounding the ligand binding pocket where the water structure near Ser810 was modified in the AZD9977 complex structure and Met777 adopted a unique conformation (Fig 3b). Met777 lies directly in between helices 3, 5 and 12 close to the activation function 2 (AF2) which is a key surface for co-regulator interactions. In the closely related glucocorticoid receptor (GR) the corresponding residue is a valine and the GR Val571Met mutation affects both ligand selectivity and transcriptional activity [49]. These observations suggest that Met777 is critically involved in mediating AZD9977 actions and mutation of Met777 will change the functional profile of AZD9977. To test this hypothesis, AZD9977 and eplerenone were tested on the MR Met777Val mutant in a reporter gene assay. AZD9977 was unable to significantly block aldosterone mediated activation and caused nearly full receptor activation of the MR Met777Val mutant in absence of aldosterone (Fig 3c). In contrast, eplerenone remained a full antagonist on the MR Met77Val mutant. The data suggest that the ligands exploit different intramolecular communication pathways to modulate the AF2 surface and to drive functional antagonism with Met777 being a key residue mediating AZD9977 effects.

In line with previous reports on MR co-regulator interactions [31, 32], we measured the binding of the MR LBD to various co-activator peptides using SPR at saturating compound conditions to characterize the structural state of the AF-2 surface. The data was analysed in relation to dexamethasone, a full agonist on MR, which was used to maintain the ligand-binding competence of the receptor during the experiments and thus served as an activity reference. Fig 3d shows that the capacity to recruit co-activator peptides reflects the functional efficacy observed in the reporter gene assay (Fig 2); the agonist aldosterone exhibits the strongest peptide binding and the full antagonist eplerenone the weakest over a range of co-activator peptides. The peptide binding caused by AZD9977 is intermediate, reflecting the partial antagonism measured for this compound.

### AZD9977 improves kidney function and histology in animal models of CKD

**Fig 4:**
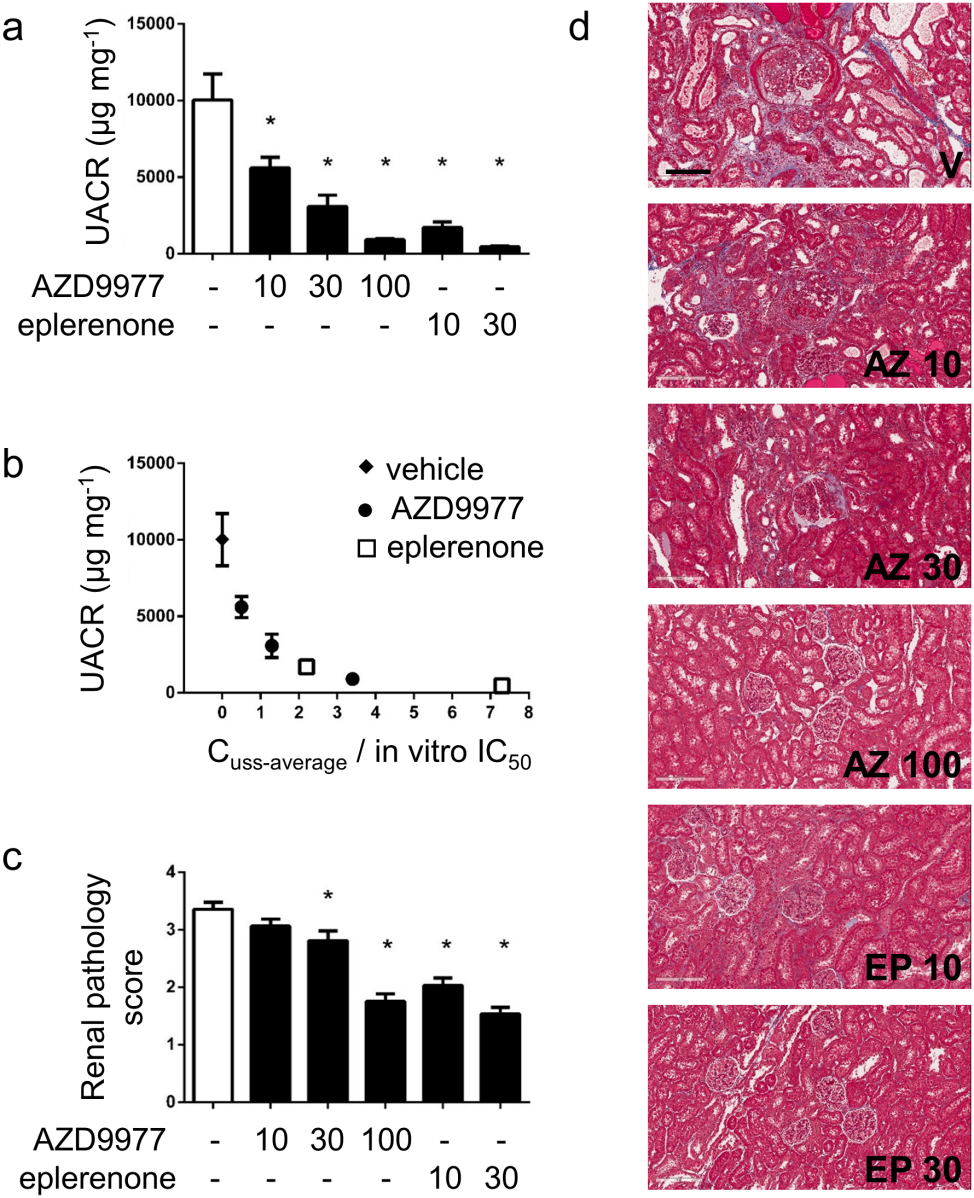
AZD9977 and eplerenone protect against aldosterone and high salt induced renal injury. Uni-nephrectomised rats with an aldosterone mini-pump and on a high salt diet were treated for 4 weeks with AZD9977 or eplerenone as food admixtures. Doses in mg kg^-1^ d^-1^ are indicated under the bars. (a) UACR was measured in urine collected for 24h at day 28. (b) UACR vs free steady average plasma drug exposure normalized to rat *in vitro* IC_50_ (C_uss_-average/*in vitro* IC_50_). Drug exposure levels correspond to the exposure levels achieved at the studied doses (a). c) Renal pathology scores (renal fibrosis and glomerular nephritis). d) Histological sections stained with PAS.Representative photomicrographs of glomeruli/tubular sections from rats treated with vehicle (V), AZD9977 at 10 (AZ 10), 30 (AZ 30) or 100 mg kg^-1^ d^-1^ (AZ 100) or eplerenone at 10 (EP 10) or 30 mg kg^-1^ d^-1^ (EP 30). The scale bar corresponds to 200 μm original size. Average +/- SEM; *n* = 7–8; *p<0.05 compared to vehicle.

The effect of AZD9977 on organ protection was assessed in uni-nephrectomised male Sprague Dawley rats administered aldosterone via osmotic minipumps and fed a high-salt diet with AZD9977 or eplerenone admixed in diet for four weeks. The model is well suited to study on-target mediated effects of MR antagonists and modulators. AZD9977 and eplerenone dose dependently reduced the UACR compared to vehicle treated animals (Fig 4a). The left shifted dose response for eplerenone compared to AZD9977 is explained by *in vitro* potency differences on the rat receptor and the achieved exposures. Applying pharmacokinetic-pharmacodynamic analysis to the data revealed that AZD9977 has the same effect on UACR reduction as eplerenone at corresponding exposure in relation to MR *in vitro* IC_50_ (Fig 4b). This implies that AZD9977 is as efficacious as full MR antagonists on renal protection, despite the partial antagonism observed in *in vitro* assays.

Reduction of the average renal pathology score was seen at the 30 and 100 mg kg^-1^d^-1^ doses of AZD9977 and the 10 and 30 mg kg^-1^ d^-1^ doses of eplerenone (Fig 4c).

**Fig 5:**
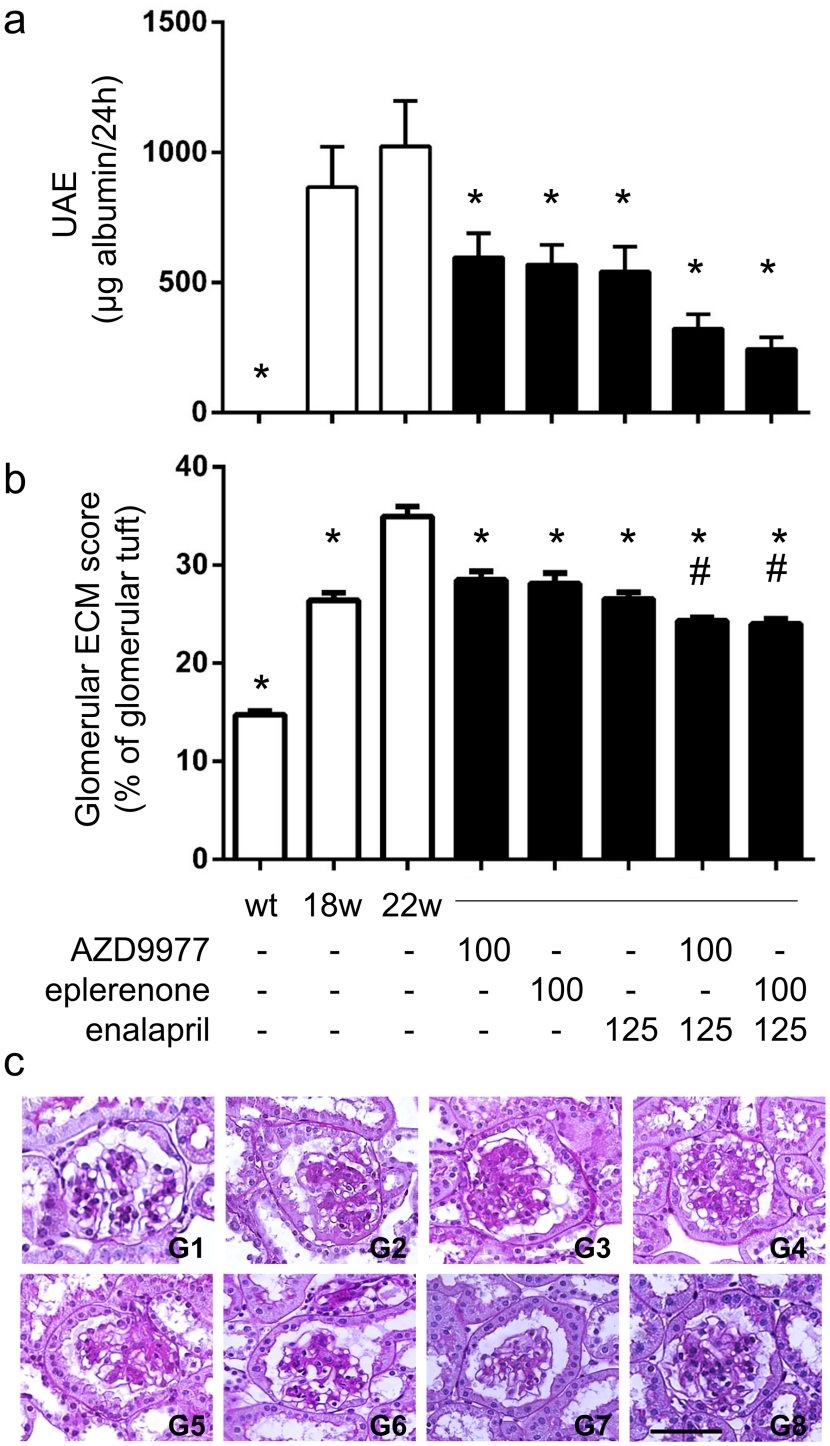
AZD9977, eplerenone and co-treatment with enalapril reduce albuminuria in diabetic kidney disease. db/db mice uni-nephrectomised at 8 weeks of age were treated from age 18w to age 22w with 100 mg kg^-1^ d^-1^ AZD9977 or 100 mg kg^-1^ d^-1^ eplerenone alone or in combination with enalapril. (a) Total urine albumine secreted over 24 hours was assessed. (b) Glomerular ECM scores were quantitated as % of glomerular tuft staining. (c) Histological sections stained with PAS. Representative photomicrographs of glomeruli from lean control mice (L), diabetic db/db mice without treatment at 18 weeks of age (w18) or at 22 weeks of age (w22) and diabetic db/db mice treated with AZD9977 (AZ), eplerenone (EP), enalapril (EN), combination of AZD9977 and enalapril (EN+AZ) and eplerenone and enalapril (EN+EP). The scale bar corresponds to 50 μm original size. Average +/- SEM; *n* = 7–9; *p<0.05 compared to vehicle at 22 weeks of age. ^#^p<0.05 compared to enalapril treatment.

Organ protective effects of AZD9977 were also studied in a disease model for diabetic nephropathy. In this model, eplerenone and enalapril have previously been shown to slow kidney deterioration in an additive manner [50]. Obese db/db mice uni-nephrectomised at 8 weeks of age developed robust kidney damage by 18 weeks of age and were then treated for a four week period. AZD9977 administered as food admixture not only stopped further disease progression but also reduced the urine albumin excretion (UAE) already established at treatment start compared to vehicle treatment (Fig 5a). Eplerenone dosed as food admixture or enalapril (ACEi) administered via the drinking water had similar effects on disease progression and intervention. Based on measured terminal plasma levels, AZD9977 and eplerenone had similar drug exposures (within a factor 2) in relation to *in vitro* IC_50_ when taken plasma protein binding into account (S2 Table). Co-administration of either AZD9977 or eplerenone with enalapril had an apparent additive effect on UAE reduction, although this reduction was not statistically significant.

At the end of the study, the glomeruli of db/db mice at 22 weeks of age demonstrated increased glomerulosclerosis with evident accumulation of PAS-positive extracellular matrix (ECM) proteins (Fig 5b and 5c). Renal pathology quantitated as glomerular ECM scoring was elevated in all obese animals, the increased injury observed from week 18 to 22 in vehicle animals was prevented by either AZD9977, eplerenone or enalapril treatment. Co-administration of enalapril with either AZD9977 or eplerenone had an additive effect on renal pathology scoring.

### AZD9977 does not affect urinary Na^+^/K^+^ acutely

**Fig 6:**
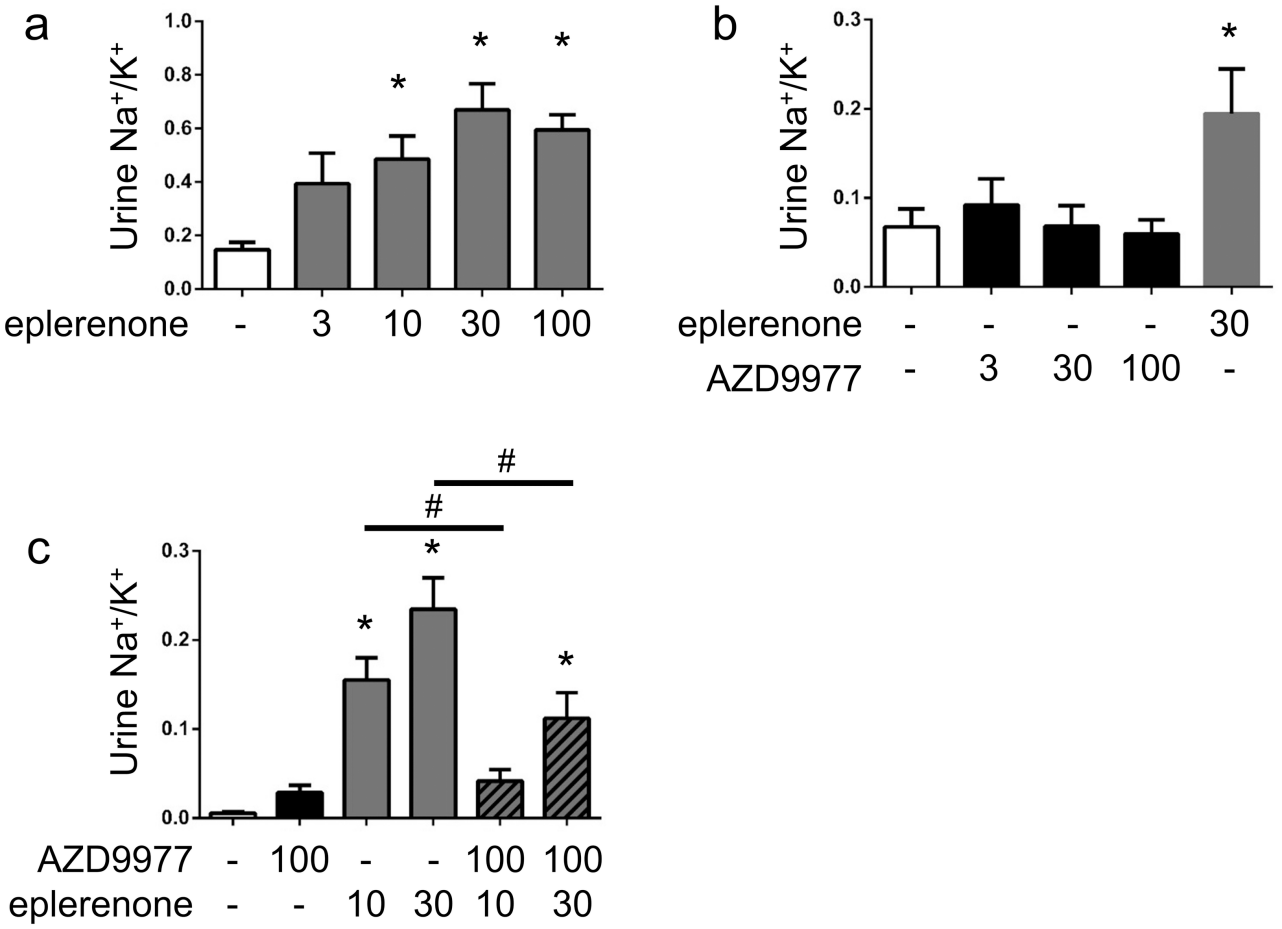
AZD9977 does not change urinary Na^+^/K^+^ ratio in rat urine electrolyte secretion model. a) Salt deprived rats were treated with increasing doses of eplerenone (a) or AZD9977 (b), urine collection for 8 hours after dose. Average +/- SEM; *n* = 8; *p<0.05 compared to vehicle. c) Salt deprived rats were treated with 10 or 30 mg kg^-1^ eplerenone in absence or presence of 100 mg kg^-1^ AZD9977, urine collection for 8 hours after dose. Average +/- SEM; *n* = 8, *p<0.05 vs vehicle. ^#^p<0.05 vs eplerenone treatment in absence of AZD9977.

The effect of AZD9977 and eplerenone on urinary Na^+^ and K^+^ excretion was studied in conscious rats placed on a low salt diet for three days to elevate endogenous aldosterone levels. In line with established MRA pharmacology, eplerenone administration caused a dose dependent elevated urine Na^+^/K^+^ ratio primarily driven by increased Na^+^ excretion (Fig 6a, S2 Fig). Interestingly, AZD9977 at equivalent exposures did not affect the urine Na^+^/K^+^ ratio (Fig 6b).

When co-administered with 10 or 30 mg kg^-1^ eplerenone, 100 mg kg^-1^ AZD9977 attenuated the Na^+^ excretion caused by eplerenone (Fig 6c). Co-administration did not alter total exposures, C_max_ or T_max_ of either eplerenone or AZD9977 (S2 Fig). The urinary Na^+^/K^+^ ratio observed at 30 mg kg^-1^ eplerenone was approximately halved by the co-administration of 100 mg kg^-1^ AZD9977, which yields an equivavlent drug exposure over IC_50_ as 30 mg kg^-1^ eplerenone (Table 3).

**Table 3:** Average drug exposure of AZD9977 and eplerenone after oral administration to salt deprived rats.

	Dose (mg kg^-1^)	C_av,in interval_ μM	Fold of *in vitro* IC_50_
AZD9977	3	0.25 (0.20; 0.32)	1.9
	30	2.5 (2.0; 3.1)	20
	100	8.2 (6.6;10.5)	65
eplerenone	3	0.41 (0.35;0.47)	8.7
	10	1.4 (1.2;1.6)	29
	30	4.1 (3.6;4.7)	86
	100	13.6 (11.8;15.6)	290

C_av,in interval_: estimated average drug exposure levels in the study interval and reported as average and 90% Confidence Interval. Unbound fraction in rat plasma (AZD9977: 57%; eplerenone: 66%) was used to convert C_av,in interval_ to free plasma levels. The free plasma levels were related to *in vitro* potencies (free plasma levels / *in vitro* IC_50_).

### Electrolyte effects in chronic models

The surprising lack of effect on urine electrolytes observed with AZD9977 in the acute experiments led us to analyse electrolytes also in the two chronic models (S1 and S2 Tables). The electrolyte effects observed in the rat model do not permit generalised conclusions regarding electrolyte handling due to the excessive sodium amounts fed in conjunction with potassium complementation in the drinking water. In the uni-nephrectomised db/db mice, total urine Na^+^ or K^+^ excretion was not affected by any of the treatments, yet urine Na^+^/K^+^ ratio was elevated in the animals eating AZD9977 or eplerenone containing food. 24 hour urine Na^+^ excretion and urine Na^+^/K^+^ ratio is substantially reduced from w18 to w22. Fractional Na^+^ excretion was elevated in the eplerenone treatment groups. AZD9977 or eplerenone treatment did not cause plasma K^+^ elevations whereas enalapril treatment did, either by itself or in combination with AZD9977 or eplerenone. Fractional K^+^ excretion was reduced in the enalapril treatment groups.

## Discussion

Multiple pre-clinical and clinical studies have shown the potential of MRAs as possible novel, efficacious treatment for CKD, which may result in both improvement of kidney function [18, 19] and a significant reduction of the high CV risk in this patient population [20]. The clinical use of MRAs in advanced CKD patients is limited in particular due to the mechanism based hyperkalemia risk. Spironolactone is in addition associated with adverse sexual side effects due to its affinity for androgen and progesterone receptors, eplerenone use is further limited by its PK properties, requiring twice daily dosing and difficult, thus expensive synthesis. Therefore there is an increasing interest to identify novel, non-steroidal MR antagonists with a differentiated side effect profile and several potent, non-steroidal MRAs are currently in clinical development [51, 52]. A reduced risk for hyperkalemia has been suggested for finerenone, the most advanced of these novel MRAs, based on differential tissue distribution into cardiac vs renal tissue [53–55].

Spironolactone and eplerenone as well as novel, non-steroidal MRAs have been selected based on their ability to acutely and dose-dependently increase urinary Na^+^/K^+^ ratio [56–60], which has been established as a quantitative, translatable, mechanism-based marker of MR antagonism [61]. The effects of MRAs on urinary electrolyte excretion are attributed to the blockade of aldosterone action in the renal distal tubule. Aldosterone stimulates urinary Na^+^ reabsorption in the principal cells of the collecting duct primarily by activation of ENaC [62], which increases the electronegativity of the lumen potential and thereby drives increased urinary K^+^ excretion [63].

Renal excretion of K^+^ accounts for >90% of total K^+^ elimination and the kidney maintains its ability to secrete K^+^ also in CKD by an adaptive increase in the secretory rate of K^+^ until GFR falls below a critical level of 15–20 mL/min/1.73 m^2^ [64]. Whilst extrarenal K^+^ adaption may play an increasing role in situations of high K^+^ intake or severe renal impairment, data on the importance of extrarenal K^+^ adaption and the role of aldosterone/MR in this process are conflicting [65, 66]. Blockade of aldosterone mediated urinary Na^+^ reabsorption by MRAs and the consequently reduced urinary K^+^ excretion is therefore considered a key driving force for the MRA mediated elevation in serum K^+^ and subsequent risk for hyperkalemia observed in man.

Since impairment of renal K^+^ excretion appears to be a key factor for the MRA mediated hyperkalemia, an MR modulator void of effects on urinary electrolyte excretion should reduce the mechanism based hyperkalemia risk of MRAs significantly. The approach taken in the present study therefore was to actively look for an MR modulator which in acute testing does not affect urinary electrolyte excretion. This resulted in the discovery of the novel MR modulator AZD9977, which combines organ protective effects with lack of acute effect on urinary electrolyte excretion. Eplerenone was chosen as comparator in all studies described due to its improved selectivity profile towards the related oxysteroid receptors and lack of active metabolites compared to spironolactone. Despite a 10–20 fold reduced receptor potency *in vitro* for eplerenone compared to spironolactone, similar clinical efficacies are achieved when doses are adjusted to account for phamacokinetic and potency differences [67].

Binding of AZD9977 to MR results in an MR conformation that is different from the one induced by full MR antagonists. This is manifested by the different interaction patterns in the ligand binding pocket and the distinct co-activator peptide recruitment pattern. Unique structural states leading to distinct receptor modulation have the potential to yield differentiated co-regulator recruitment and physiological readouts [68]. The concept of using a partial agonist to mechanistically separate the functional response in different cells and tissues is well established for the estrogen receptor [69]. However, while the biophysical and functional data supports that AZD9977 is well positioned to exhibit tissue specific effects, additional studies in different cell types with different co-regulator backgrounds are required to build a complete mechanistic understanding of the AZD9977 molecular mode of action.

With the potential limitation that only male animals were studied, AZD9977 preserves renal function and structural integrity with similar efficacy as eplerenone in two rodent models of chronic kidney disease. In contrast, AZD9977 did not increase the urinary Na^+^/K^+^ ratio in acute testing, the hallmark feature of traditional MR antagonists. The effect of eplerenone on urinary Na^+^/K^+^ ratio was attenuated by co-administration of AZD9977 and approximately halved at equivalent exposures over IC_50_, implying a competitive interaction at the receptor. This suggests that AZD9977 affects aldosterone induced Na^+^ reabsorption in a fundamentally different way than eplerenone.

The acute, marked effect on urine Na^+^ excretion seen with MRA’s in rodent species readily translates into observations in man [61] and as discussed above provides a mechanistic explanation of MRA elicited plasma K^+^ increases, despite the fact that plasma K^+^ elevations are only observed after several days of treatment in man. In the current study, 4 weeks treatment of uni-nephrectomised db/db mice with eplerenone or AZD9977 did not cause plasma K^+^ elevations, in line with previous observations [50, 70] where MRA treatment did not effect plasma K^+^ in rodents.

In contrast to previously published data [61] we observe a small yet statistically significant increase of urine Na^+^/K^+^ after 4 weeks feeding of eplerenone or AZD9977 containing food to uni-nephrectomised db/db mice. Examining the individual electrolytes, however, there was no difference in 24 h excretion of Na^+^ or K^+^ between treatment groups. Notably, the urine Na^+^ excretion and hence Na^+^/K^+^ was higher before treatment start (w18) than after (w22) and the treatment related changes were within the range of the w18 and w22 vehicle groups. The apparent effect on urine Na^+^/K^+^ ratio after chronic treatment in this study does not translate into plasma K^+^ elevations. In contrast to a previous study in this model [50], chronic enalapril treatment here led to plasma K^+^ elevations, hence reduced fractional K^+^ excretion is observed in enalapril treated animals. The inconsistent urine and plasma electrolyte effects after chronic ACEi and MRA treatment in db/db mice confirm that rodents are challenging to study electrolyte handling under chronic conditions and suggest that the potential differentiation regarding plasma K^+^ elevations between AZD9977 and eplerenone must be studied in more sensitive models.

Our data highlight that the beneficial effects on renal and cardiac dysfunction seen with MR antagonists cannot solely be attributed to Na^+^ loss and concomitant volume effects. This is in line with previous pre-clinical and clinical studies comparing effects of MRAs with calcium channel blockers or loop diuretics. Despite similar blood pressure lowering effects, only MRAs significantly attenuate proteinuria both pre-clinically and clinically and were shown to prevent kidney damage in pre-clinical models [4, 21]. In the diabetic nephropathy disease model, AZD9977 and eplerenone were as efficacious as enalapril which is the clinical standard of care. Interestingly, co-treatment with enalapril with either eplerenone or AZD9977 yielded further improvement on renal histopathology compared to standard of care alone. The effects on top of enalapril hold great promise particularly for AZD9977. Aldosterone breakthrough after prolonged ACE inhibition may be treated by co-administering an MRA. However, since ACE inhibition already carries a risk for elevated plasma K^+^, adding a traditional MRA further increases the risk for hyperkalemia [71]. AZD9977, with its potential reduced risk to cause hyperkalemia, is therefore well positioned as an add-on treatment to standard of care also in CKD.

In conclusion, AZD9977 is a novel and selective, non-steroidal MR modulator, which in pre-clinical testing separates organ protective effects from acute effects on urinary electrolyte excretion, which predicts a reduced risk of hyperkalemia. Structural data and the differential co-factor peptide recruitment profile of AZD9977 suggest that the unique pharmacological profile of AZD9977 may be a consequence of the distinct interaction pattern between AZD9977 and MR. Further in depth mode of action studies are required to pinpoint the molecular pathways and interactions of AZD9977 leading to the observed differences in urinary electrolyte excretion and to identify common pathways leading to organ protection. If the observed pre-clinical profile of AZD9977 is shown to translate into human, AZD9977 has the potential to deliver a safe and efficacious treatment to patients with high risk for hyperkalemia, in particular CKD patients.

## Supporting information

S1 Table Uni-nephrectomised rats on high salt diet and aldosterone infusion, general measures. (PDF)

S2 Table Uni-nephrectomised db/db mice, general measures. (PDF)

S1 Fig Individual electrolytes in acute testing. (PDF)

S2 Fig Exposure levels of AZD9977 and eplerenone after co-administration in acute model. (PDF)
