# Supplementary material for: Preclinical pharmacology of AZD9977: A novel mineralocorticoid receptor modulator separating organ protection from effects on electrolyte excretion
Source: PLoS One. 2018 Feb 23;13(2):e0193380. doi: 10.1371/journal.pone.0193380 (PMC5825103; doi:10.1371/journal.pone.0193380)
Supplement: S1 Table — (PDF) [file pone.0193380.s001.pdf]

S1 Table. Uni-nephrectomised rats on high salt diet and aldosterone infusion, general measures

		Vehicle (*n*=7)	AZD9977 10 mg kg^{-1} d^{-1} (*n*=8)	AZD9977 30 mg kg^{-1} d^{-1} (*n*=8)	AZD9977 100 mg kg^{-1} d^{-1} (*n*=7)	eplerenone 10 mg kg^{-1} d^{-1} (*n*=8)	eplerenone 30 mg kg^{-1} d^{-1} (*n*=7)
**BW (g)**	start	365 ± 8	361 ± 8	363 ± 5	365 ± 8	361 ± 4	361 ± 5
	end	513 ± 14	505 ± 11	514 ± 13	516 ± 20	519 ± 14	520 ± 15
**UACR (µg mg^{-1})**	start	15 ± 2	20 ± 7	13 ± 3	13 ± 2	11 ± 2	10 ± 2
	2w	7402 ± 2109	2133 ± 705 *	1200 ± 569 *	148 ± 34 *	408 ± 173 *	88 ± 23 *
	end	10027 ± 1704	5604 ± 689 *	3076 ± 756 *	900 ± 83 *	1689 ± 394 *	456 ± 66 *
**u-Na^+/K^+**	end	2.27 ± 0.04	2.34 ± 0.04	2.53 ± 0.04*	2.71 ± 0.05*	2.51 ± 0.05*	2.77 ± 0.03*
**u-Na^+ (mmol/24h)**	end	26.2 ± 1.2	23.8 ± 1.0	26.6 ± 0.9	24.7 ± 1.1	25.4 ± 1.1	26.7 ± 0.9
**u-K^+ (mmol/24h)**	end	11.6 ± 0.6	10.2 ± 0.4	10.6 ± 0.5	9.2 ± 0.5 *	10.1 ± 0.5	9.6 ± 0.3 *
**p-K^+ (mM)**	end	4.8 ± 0.2	4.9 ± 0.2	5.0 ± 0.3	5.2 ± 0.1	4.8 ± 0.2	5.1 ± 0.2
**FE K^+ (%)**	end	33.9 ± 2.2	28.0 ± 2.6	27.2 ± 1.2*	20.1 ± 0.7*	25.0 ± 1.3*	23.0 ± 1.0*
**p-crea (µg mL^{-1})**	end	3.5 ± 0.2	3.4 ± 0.3	3.4 ± 0.2	2.9 ± 0.1	2.9 ± 0.1	3.2 ± 0.2
**WI (mL/24h)**	end	194 ± 13	171 ± 14	159 ± 8	121 ± 10*	144 ± 7*	129 ± 6*
**$C_{ss, average}$ (µM)**			0.07 ± 0.01	0.17 ± 0.03	0.46 ± 0.06	0.09 ± 0.02	0.30 ± 0.09

BW: body weight; UACR: urinary albumine/creatinine ratio; u-Na^+/K^+ : urine Na^+/K^+ ratio; u-Na^+: amount Na^+ excreted in urine over 24h; u-K^+: amount K^+ excreted in urine over 24h; p-K^+: plasma K^+; FE K^+: fractional K^+ excretion; p-crea: plasma creatinine; WI: water intake; $C_{ss, average}$: estimated average drug exposure levels, unbound fraction in plasma (AZD9977 57%, eplerenone 66%) was used to convert $C_{ss,average}$ to free plasma levels. Average ± SEM. * $p<0.05$ vs vehicle group.
