# Supplementary material for: Preclinical pharmacology of AZD9977: A novel mineralocorticoid receptor modulator separating organ protection from effects on electrolyte excretion
Source: PLoS One. 2018 Feb 23;13(2):e0193380. doi: 10.1371/journal.pone.0193380 (PMC5825103; doi:10.1371/journal.pone.0193380)
Supplement: S2 Table — (PDF) [file pone.0193380.s002.pdf]

S2 Table. Uni-nephrectomised db/db mice, general measures

		lean vehicle (*n*=9)	obese w18 (*n*=10)	obese vehicle (*n*=9)	obese AZD9977 (*n*=8)	obese eplerenone (*n*=9)	obese enalapril (*n*=9)	obese enalapril + AZD9977 (*n*=7)	obese enalapril + eplerenone (*n*=9)
**BW (g)**	w18	27.9 ± 0.5	49.7 ± 2.5	50.3 ± 2.2	48 ± 2.3	49.1 ± 2	47.5 ± 1.5	50.2 ± 2.2	49.4 ± 2.1
	w22	29.1 ± 0.5*		46.6 ± 2.5	41.4 ± 1.9	42.5 ± 1.8	39.3 ± 1.8	39.9 ± 2.3	38.3 ± 3.5*
**BG (mM)**	w18	8.7 ± 0.5	>29.4	>29.4	>29.4	>29.4	>29.4	>29.4	>29.4
	w22	9.1 ± 0.5		>29.4	>29.4	>29.4	>29.4	>29.4	>29.4
**HbA1c (%)**	w18	4.7 ± 0.1	>12	>12	>12	>12	>12	>12	>12
	w22	4.7 ± 0.1		>12	>12	>12	>12	>12	>12
**UAE (µg 24h^{-1})**	w18	3 ± 1	867 ± 155	907 ± 138	797 ± 169	892 ± 186	958 ± 184	1055 ± 296	832 ± 129
	w22	1 ± 0		1023 ± 175	588 ± 80 *	569 ± 76 *	583 ± 97 *	356 ± 52 *	244 ± 51 *
**u-Na^+/K^+**	end		0.53 ± 0.01*	0.39 ± 0.01	0.47 ± 0.01*	0.48 ± 0.02*	0.40 ± 0.01	0.47 ± 0.01*	0.50 ± 0.02*
**u-Na^+ (mmol/24h)**	end		0.52 ± 0.04*	0.37 ± 0.02	0.45 ± 0.05	0.40 ± 0.05	0.36 ± 0.03	0.39 ± 0.03	0.39 ± 0.04
**u-K^+ (mmol/24h)**	end		0.98 ± 0.06	0.94 ± 0.04	0.97 ± 0.10	0.85 ± 0.10	0.90 ± 0.07	0.84 ± 0.07	0.80 ± 0.10
**p-K^+ (mM)**	end	3.2 ± 0.1		3.6 ± 0.1	3.9 ± 0.2	3.8 ± 0.1	4.4 ± 0.1*	4.8 ± 0.2*	4.7 ± 0.3*
**FE K^+ (%)**	end			20.5 ± 1.2[#]	20.1 ± 0.9[#]	22.3 ± 1.0[#]	15.6 ± 0.4*	15.6 ± 0.8*	17.8 ± 1.0
**FE Na^+ (%)**	end			0.19 ± 0.01	0.24 ± 0.01	0.27 ± 0.02*	0.18 ± 0.01	0.24 ± 0.02	0.27 ± 0.02*
**p-crea (µg mL^{-1})**	end	9.9 ± 0.5*	5.1 ± 0.2	5.3 ± 0.3	5.8 ± 0.2	5.9 ± 0.2	5.0 ± 0.2	5.6 ± 0.3	6.4 ± 0.6
**C_{drug} (µM)**					0.35 ± 0.04	0.15 ± 0.02		0.27 ± 0.05	0.12 ± 0.03
**Fold IC_{50}**					2.4	1.1		1.9	0.9

BW: body weight; BG: blood glucose; UAE: urinary albumine excretion; u-Na^+/K^+ : urine Na^+/K^+ ratio; u-Na^+: amount Na^+ excreted in urine over 24h; u-K^+: amount K^+ excreted in urine over 24h; p-K^+: plasma K^+; FE K^+: fractional K^+ excretion; FE Na^+: fractional Na^+ excretion; p-crea: plasma creatinine; C_{drug}: terminal drug exposure levels. Unbound fraction in plasma (AZD9977 55%, eplerenone 69%) was used to convert C_{drug} to free plasma levels; Fold IC_{50}: free plasma levels related to *in vitro* potencies (free plasma levels / *in vitro* IC_{50}). Average ± SEM. *$p<0.05$ vs obese vehicle at w22, $^{\#}p<0.05$ vs enalapril.
