# Supplementary material for: Preclinical pharmacology of AZD9977: A novel mineralocorticoid receptor modulator separating organ protection from effects on electrolyte excretion
Source: PLoS One. 2018 Feb 23;13(2):e0193380. doi: 10.1371/journal.pone.0193380 (PMC5825103; doi:10.1371/journal.pone.0193380)
Supplement: S1 Fig — (PDF) [file pone.0193380.s003.pdf]

S1 Fig. Individual electrolytes in acute testing

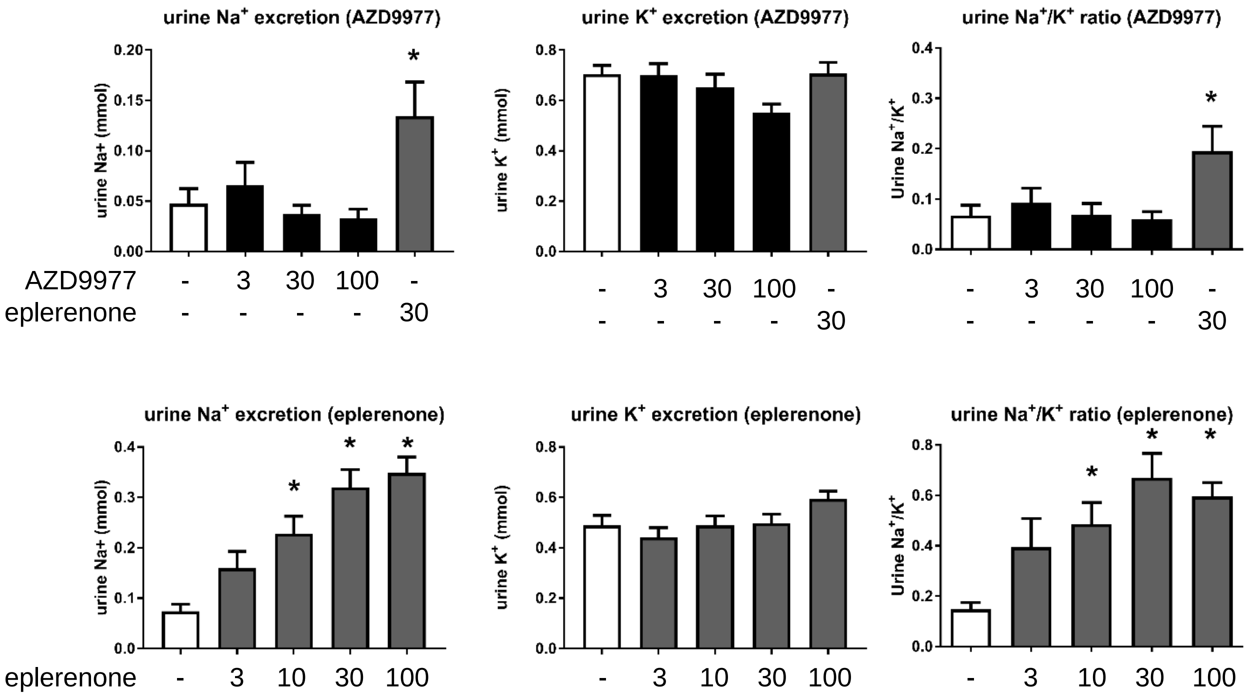

Excreted amounts of Na^+ and K^+ over 8 hrs. Salt deprived rats were treated with increasing doses of AZD9977 or eplerenone (mg kg^{-1} as indicated), urine collection for 8 hours after dose. Average +/- SEM; n =8; *$p<0.05$ compared to vehicle.
