# Supplementary material for: Preclinical pharmacology of AZD9977: A novel mineralocorticoid receptor modulator separating organ protection from effects on electrolyte excretion
Source: PLoS One. 2018 Feb 23;13(2):e0193380. doi: 10.1371/journal.pone.0193380 (PMC5825103; doi:10.1371/journal.pone.0193380)
Supplement: S2 Fig — (PDF) [file pone.0193380.s004.pdf]

S2 Fig. Co-administration did not alter total exposures of either eplerenone or AZD9977

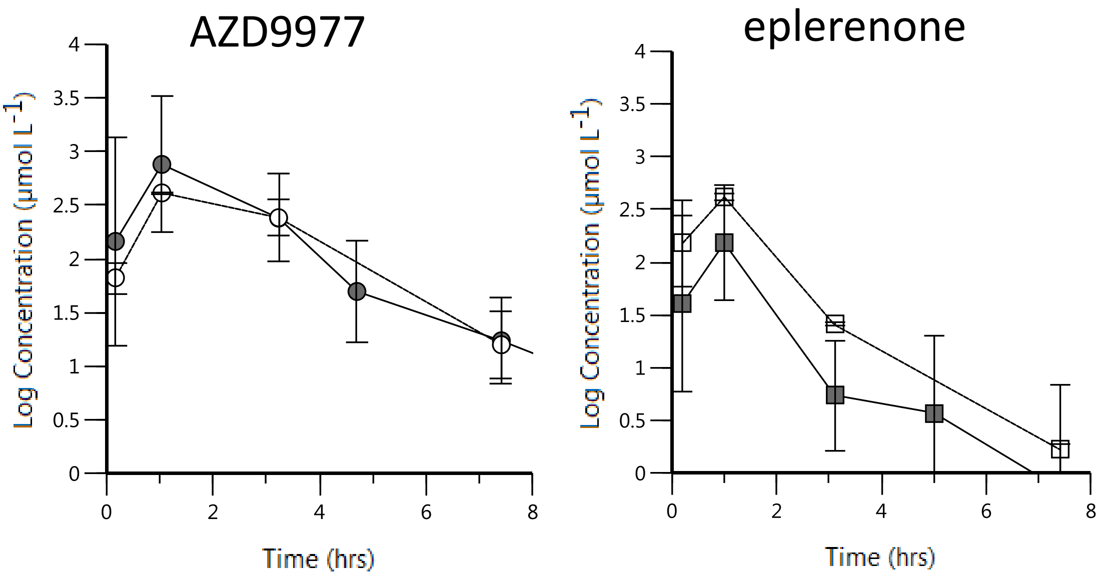

Left graph: AZD9977 concentration vs time after single oral dose administration of 100 mg kg^{-1} AZD9977 (solid circles) or 100 mg kg^{-1} AZD9977 plus 30 mg kg^{-1} eplerenone (open circles).
Right graph: eplerenone concentration vs time after single oral dose administration of 30 mg kg^{-1} eplerenone (solid squares) or 30 mg kg^{-1} eplerenone plus 100 mg kg^{-1} AZD9977 (open squares). Log Mean ± log SD. N=2-9 per time point.

Co-administration did not alter total exposures of either eplerenone or AZD9977: when 100 mg kg^{-1} AZD9977 was administered alone or co-administered with eplerenone 30 mg kg^{-1} the median $AUC_{0\text{-}8hrs}$ for AZD9977 was 66 µM x hrs (25th; 75th percentile: 44;116) and 66 µM x hrs (25th; 75th percentile: 60; 72), respectively. When 30 mg kg^{-1} eplerenone was administered alone or co-administered with AZD9977 100 mg kg^{-1} the median $AUC_{0\text{-}8hrs}$ for eplerenone was 26 µM x hrs (25th; 75th percentile: 14;41), and 40 µM x hrs (25th; 75th percentile: 37; 43), respectively. AUC was calculated using a non-compartmental analysis with linear trapezoidal and linear interpolation method, as implemented in Phoenix® WinNonlin® version 6.4, NMLE version 1.3 (Certara USA, Inc., Princeton, NJ).
